# Single-cell genomics reveals features of a *Colwellia* species that was dominant during the Deepwater Horizon oil spill

**DOI:** 10.3389/fmicb.2014.00332

**Published:** 2014-07-08

**Authors:** Olivia U. Mason, James Han, Tanja Woyke, Janet K. Jansson

**Affiliations:** ^1^Department of Earth, Ocean and Atmospheric Science, Florida State UniversityTallahassee, FL, USA; ^2^Department of Energy Joint Genome InstituteWalnut Creek, CA, USA; ^3^Lawrence Berkeley National Laboratory, Earth Sciences Division, Ecology DepartmentBerkeley, CA, USA; ^4^Department of Plant and Microbial Biology, University of CaliforniaBerkeley, CA, USA

**Keywords:** DWH oil spill, *Colwellia*, single-cell genomics, deep-sea plume, hydrocarbon degradation, bacteria

## Abstract

During the Deepwater Horizon (DWH) oil spill in the Gulf of Mexico a deep-sea hydrocarbon plume developed resulting in a rapid succession of bacteria. *Colwellia* eventually supplanted *Oceanospirillales*, which dominated the plume early in the spill. These successional changes may have resulted, in part, from the changing composition and abundance of hydrocarbons over time. *Colwellia* abundance peaked when gaseous and simple aromatic hydrocarbons increased, yet the metabolic pathway used by *Colwellia* in hydrocarbon disposition is unknown. Here we used single-cell genomics to gain insights into the genome properties of a *Colwellia* enriched during the DWH deep-sea plume. A single amplified genome (SAG) of a *Colwellia* cell isolated from a DWH plume, closely related (avg. 98% 16S rRNA gene similarity) to other plume *Colwellia*, was sequenced and annotated. The SAG was similar to the sequenced isolate *Colwellia psychrerythraea* 34H (84% avg. nucleotide identity). Both had genes for denitrification, chemotaxis, and motility, adaptations to cold environments and a suite of nutrient acquisition genes. The *Colwellia* SAG may be capable of gaseous and aromatic hydrocarbon degradation, which contrasts with a DWH plume *Oceanospirillales* SAG which encoded non-gaseous *n*-alkane and cycloalkane degradation pathways. The disparate hydrocarbon degradation pathways are consistent with hydrocarbons that were abundant at different times in the deep-sea plume; first, non-gaseous *n*-alkanes and cycloalkanes that could be degraded by *Oceanospirillales*, followed by gaseous, and simple aromatic hydrocarbons that may have been degraded by *Colwellia*. These insights into the genomic properties of a *Colwellia* species, which were supported by existing metagenomic sequence data from the plume and DWH contaminated sediments, help further our understanding of the successional changes in the dominant microbial players in the plume over the course of the DWH spill.

## Introduction

The Deepwater Horizon (DWH) oil spill from April to July 2010 was unprecedented due to the extreme depth (1500 m below sea-level; mbsl) and low temperature (4°C), at which it took place. *Colwellia* species bloomed in the deep-sea hydrocarbon plume, that formed at 1100 mbsl, in early June, 2010 (Valentine et al., 2010; Redmond and Valentine, 2012) after partial capture of the oil began (Dubinsky et al., 2013). At this time the unmitigated flow of oil ceased, and cycloalkanes and non-gaseous *n*-alkanes, which were dominant until that point, decreased in concentration (Dubinsky et al., 2013). Concomitantly, the concentration of natural gases and simple aromatics increased (Dubinsky et al., 2013). The change in hydrocarbon composition and abundance resulting from partial capture was mirrored by differences in microbial community composition, with a shift in dominance of *Oceanospirillales* to *Colwellia* (Dubinsky et al., 2013). Mason et al. (2012) analyzed *Oceanospirillales* single amplified genomes (SAG) from the deep-sea plume and reported the genome encoded pathways for cyclohexane and non-gaseous *n*-alkane degradation. These data helped to explain the dominance of *Oceanospirillales* when cycloalkanes and non-gaseous *n*-alkanes were abundant, and its subsequent decline when these hydrocarbon constituents decreased. The later appearance of *Colwellia* in the spill history could be due to its ability to degrade other hydrocarbon constituents in the oil. Microcosm experiments established that *Colwellia* species were capable of degrading hydrocarbons originating from the oil spill at 4°C (Baelum et al., 2012; Redmond and Valentine, 2012). Specifically, Redmond and Valentine (2012) reported that *Colwellia* species from the deep-sea plume incorporated labeled ethane, propane, and benzene. The ability to degrade ethane and propane, for example, provides clues as to why *Colwellia* appears to have increased in abundance when the concentration of these and other gases increased in June 2010.

Hydrocarbon degradation by cultured *Colwellia* has not previously been reported (e.g., Bowman et al., 1997; Methé et al., 2005). For example, *Colwellia psychrerythraea* 34H, cultivated from Arctic marine sediments, is psychrophilic, with optimal growth at −1°C to 10°C (Huston, 2003) was not reported to degrade hydrocarbons. The genome of *C. psychrerythraea* provides insights into the adaptations that enable it to be active at such low temperatures, such as changes to cell membrane fluidity and the use of compounds that provide cryotolerance (Methé et al., 2005). Further, this genome provides a platform for comparison to *Colwellia* that were enriched during the DWH oil spill. Although the possible role of *C. psychrerythraea* in bioremediation by aromatic, or C_1_ contaminant degradation was inferred, the specific contaminants and the pathways catalyzing these reactions have not yet been elucidated (Methé et al., 2005). Thus it remains unresolved as to how *Colwellia* species that were identified during the DWH spill are capable of growth with ethane, propane, and benzene (Redmond and Valentine, 2012), polycyclic aromatic hydrocarbons (PAH) (Gutierrez et al., 2013), or MC252 crude oil constituents (Baelum et al., 2012). Here our aim was to use single-cell genomics to gain a better understanding of the genomic properties of a deep-sea *Colwellia* species that enabled it to bloom during the oil spill. Specifically, we present a genome analysis of a *Colwellia* single-cell isolated directly from the deep-sea plume described in Hazen et al. (2010) and Mason et al. (2012).

## Materials and methods

### Single-cell sorting, whole-genome amplification, and screening

Cells were collected following the clean sorting procedures detailed by Rodrigue et al. (2009). Briefly, single cells from the proximal plume water sample, collected on May 29, 2010 from 1207 mbsl (described in Mason et al., 2012), were sorted by the Cytopeia Influx Cell Sorter (BD Biosciences, Franklin Lakes, NJ) into three 96-well plates containing 3 μ l of ultraviolet-treated TE. The cells were stained with SYBR Green I (Invitrogen, Carlsbad, CA) and illuminated by a 488-nm laser (Coherent Inc., Santa Clara, CA). As described by Woyke et al. (2011) the sorting window was based on the size determined by side scatter and green fluorescence (531/40 bp filter). Single cells were lysed for 20 min at room temperature using alkaline solution from the Repli-G UltraFast Mini Kit (Qiagen, Valencia, CA) according to the manufacturer's instructions. After neutralization, the samples were amplified using the RepliPHI Phi29 reagents (Epicenter Biotechnologies, Madison, WI). Each 50-μ l reaction contained Phi29 Reaction Buffer (1 × final concentration), 50 μ M random hexamers with the phosphorothioate bonds between the last two nucleotides at the 3′ end (IDT, Coralville, IA), 0.4 mM dNTP, 5% DMSO (Sigma, St Louis, MO), 10 mM DTT (Sigma), 100 U Phi29 and 0.5 mM Syto 13 (Invitrogen). A mastermix of multiple displacement amplification (MDA) reagents minus the Syto 13 sufficient for a 96-well plate was ultraviolet-treated for 60 min for decontamination. Syto 13 was then added to the mastermix, which was added to the single cells for real-time MDA on the Roche LightCycler 480 for 17 h at 30°C. All steps of single-cell handling and amplification were performed under most stringent conditions to reduce the introduction of contamination. Single-cell MDA products were screened using Sanger sequencing of 16S rRNA gene amplicons derived from each MDA product. Two single-cell MDA products were identified as *Colwellia*, one of which was selected for shotgun sequencing and analysis based on the clean appearance of the 16S rRNA gene Sanger sequencing electropherogram.

### Single-cell illumina sequencing, quality control, and assembly

Single-cell amplified DNA of *Colwellia* was used to generate normalized, indexed Illumina libraries. Briefly, 3 μ g of MDA product was sheared using the Covaris E210 (Covaris, Woburn, MA) with the following protocol: 10% duty cycle, intensity 5 and 200 cycles per burst for 6 min per sample. The fragmented DNA was purified using QIAquick columns (Qiagen) according to the manufacturer's instructions. The sheared DNA was end-repaired, A-tailed and ligated to the Illumina adaptors according to the Illumina standard paired-end protocol. The ligation product was purified using AMPure SPRI beads, then underwent normalization using the Duplex-Specific Nuclease Kit (Axxora, San Diego, CA). The normalized libraries were then amplified by PCR for 12 cycles using a set of two indexed primers and the library pool was sequenced on one lane of an Illumina HiSeq 2000 sequencer according to the manufacturer's protocols (run mode 2 × 76 bp). To ensure that the single-amplified genome was free of contamination, the Illumina sequence data was quality controlled using GC content and blast analysis (for details, see supplemental material).

### Bioinformatics

The 16S rRNA gene sequence of the *Colwellia* SAG was compared to 16S rRNA gene datasets in plume samples and stable isotope probing (SIP) experiments (Valentine et al., 2010; Mason et al., 2012; Redmond and Valentine, 2012). SAG reads were assembled using SPAdes 2.4.0 (Bankevich et al., 2012) with the following parameters: –sc –careful –m 40 –12. The single copy gene analysis was carried out according to Rinke et al. (2013). Unassembled metagenome and metatranscriptome reads from deep-sea plume samples presented by Mason et al. (2012) (available at http://mason.eoas.fsu.edu) and metagenome reads from surface sediments (http://mason.eoas.fsu.edu and MG-RAST IDs 4510162.3-4510175.3), several of which were contaminated during the DWH spill (Mason et al., 2014), were mapped against the assembled *Colwellia* SAG using Bowtie2 (Langmead and Salzberg, 2012) with default parameters. The SAG assembly was annotated using CAMERA 2.0 (Sun et al., 2011). The reads that went into the SAG assembly were compared to *C. psychrerythraea* (Methé et al., 2005) using PAUDA (Huson and Xie, 2014), with a bit score of ≥40 serving as the cutoff. JSpecies (Richter and Rosselló-Móra, 2009) was used to compute the average nucleotide identity between the *Colwellia* SAG assembly and *C. psychrerythraea*. The average was computed by using values greater than 70%. The lowest observed value that was used in the calculation was 70.34% and the highest was 100%. Additionally, the reads that went into the *Colwellia* SAG assembly were also compared to a database of genes involved in hydrocarbon degradation (bit score of ≥40 cutoff), similar to the methodology described by Mason et al. (2012) with PAUDA (Huson and Xie, 2014). Last, the *Colwellia* SAG assembly was compared (blastx) to gene products of the BMO operon of *Thauera butanivorans* (NCBI locus AY093933). Contigs with *bmoR* were also compared to the non-redundant protein sequences (nr) in GenBank using blastx. The *Colwellia* SAG raw reads and assembly are available at: http://mason.eoas.fsu.edu, as are the *Oceanospirillales* SAG reads and assemblies from Mason et al. (2012).

## Results and discussion

### Genomic features

More than 38 million reads, representing 587X coverage were obtained (assuming a genome size of 5.0 Mb). The *Colwellia* SAG assembly resulted in 141 contigs with a total assembly size of 1.3 Mb. The estimated genome recovery was 30%, based on single-copy gene analysis (Supplementary Table 1). Average G + C content was 37.5% (Supplementary Results), compared to 37.9% for *C. psychrerythraea* (Methé et al., 2005). A total of 1163 open reading frame(s) were identified. In the assembled *Colwellia* SAG 11 rRNA operons and 8 tRNAs were present. All annotated COGs (CAMERA) of the *Colwellia* SAG assembly are shown in Supplementary Table 2. The average nucleotide identity when comparing the *Colwellia* SAG to *C. psychrerythraea* was 84%.

### SAG taxonomy

**Figure 1:**
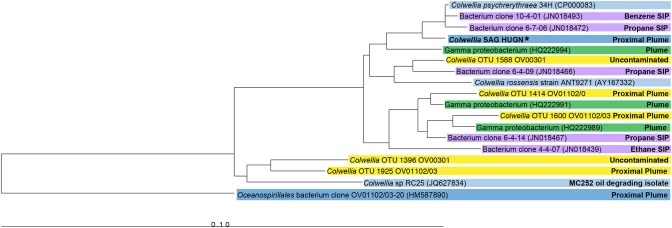
**Neighbor-joining phylogenetic tree of 16S rRNA genes of the *Colwellia* SAG and close relatives**. Sequences from a particular study are highlighted with the same color. Information about the environment from which a particular microorganism was found is indicated in the figure. The *Colwellia* SAG is in bold text and indicated by an asterisk.

The SAG selected for Illumina sequencing, *Colwellia* SAG HUGN, was closely related to *C. psychrerythraea* based on its 16S rRNA gene sequence (99% similar). This clade also includes highly similar *Colwelli*a sequences retrieved from the DWH plume (average 98%; Valentine et al., 2010; Mason et al., 2012) and by stable isotope probing analysis (97–99%; Redmond and Valentine, 2012) (Figure 1). Although the SAG was very similar to the *Colwellia* sequences identified *in situ* in the plume sample, *Colwellia* was not abundant early in the spill history at the time the sample was obtained (Mason et al., 2012). The SAG was also highly similar to *Colwellia* species found in the June 2010 plume samples (Figure 1), in which *Colwellia* had a relative abundance of >60% of the community (Valentine et al., 2010).

### Hydrocarbon degradation

**Figure 2:**
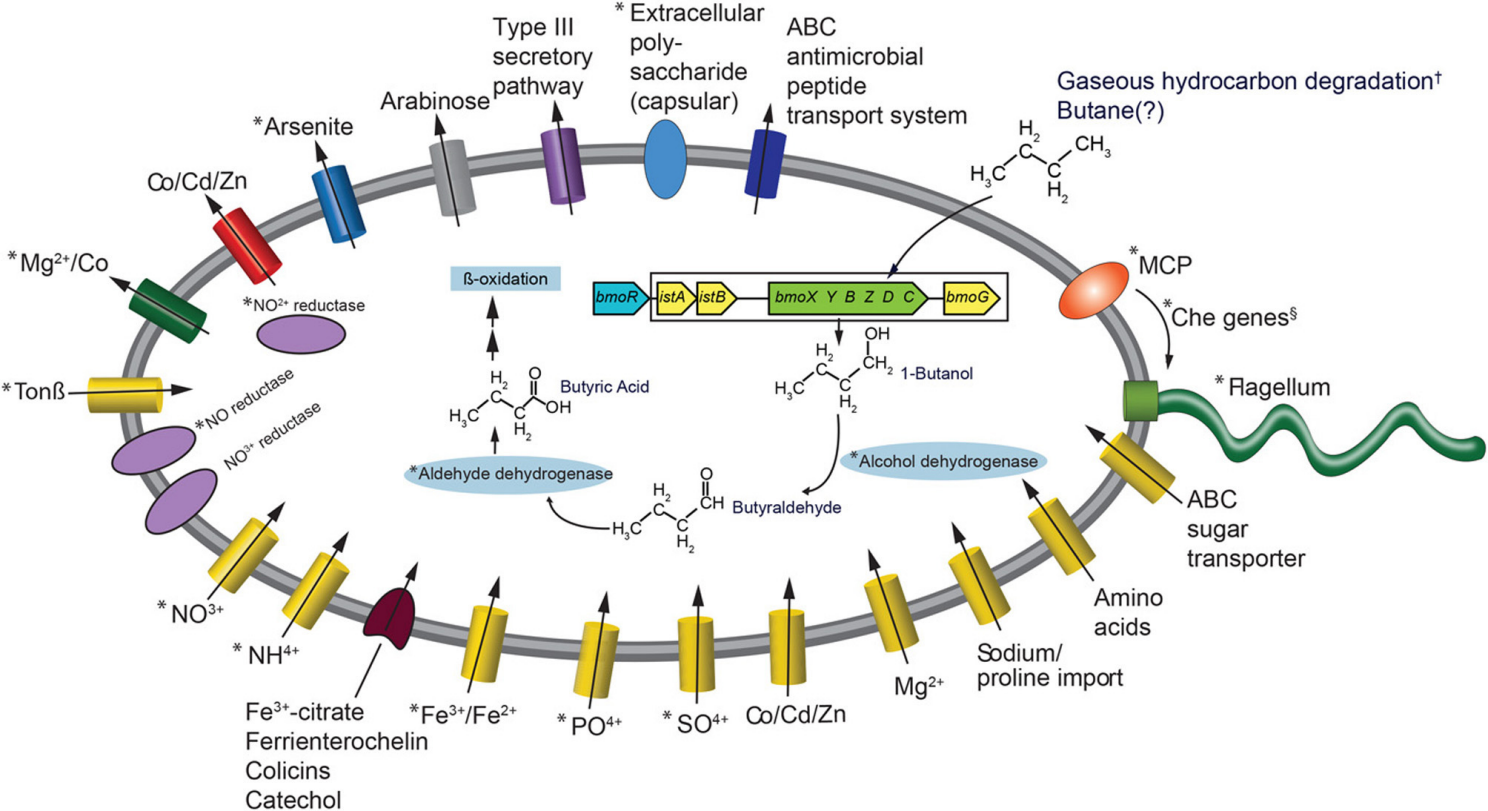
**Metabolic reconstruction of the *Colwellia* SAG**. An asterisk indicates *Colwellia* SAG reads with ≥40 bit score to *C. psychrerythraea*. The BMO operon and adjacent genes (from Kurth et al., 2008, see Figure 1A) are color coded: blue indicates *Cowellia* SAG. Additionaly, those genes from the DWH plume and sediment metagenomes (Mason et al., 2012, 2014, respectively) are enclosed by rectangle. Genes shown in yellow indicate DWH plume, those in green indicate sediment metagenomes. These genes were not directly ascribed to the *Colwellia* SAG described herein. An † indicates that the direct evidence for gaseous hydrocarbon degradation by plume *Colwellia* was provided by Redmond and Valentine (2012), not from the *Colwellia* SAG annotation. The *Colwellia* SAG had the full suite of genes coding for chemotaxis including Che genes, indicated by an §. Che genes include CheA, CheC, CheY, and CheZ.

The most frequently observed genes in the reads that went into the *Colwellia* SAG assembly were aldehyde dehydrogenase, alcohol dehydrogenase, enoyl-CoA hydratase, *bmoR*, 2-hydroxymuconic semialdehyde dehydrogenase and 4-cresol dehydrogenase. Generally, these genes could be part of several different metabolic pathways, but the observed *bmoR*, which was highly similar (avg. similarity was 74%) to that of *Pseudomonas butanovora* [subsequently renamed *Thauera butanivorans* (Dubbels et al., 2009)] suggested that it may play a similar functional role in the *Colwellia* species described here. *T. butanivorans* (Dubbels et al., 2009), isolated from activated sludge from an oil refining plant (Takahashi, 1980), is able to grow on C_2_–C_9_ alkanes (Dubbels et al., 2009) using the following pathway: Butane (BMO)→ 1-Butanol (alcohol dehydrogenase)→ Butyraldehyde (aldehyde dehydrogenase)→ Butyric Acid (further metabolized as fatty acid) (Arp, 1999). Although a butane monooxygenase was not identified in the *Colwellia* SAG presented here, *T. butanivorans*' *bmoR*, which encodes a transcriptional regulator (BmoR) for BMO, and is located upstream of the BMO operon (Kurth et al., 2008), was identified (Figure 2). A total of 758 raw reads were annotated as *bmoR* in the SAG using blastx. The average similarity was 74% to *T. butanivorans bmoR* (bit score ≥ 40). In a separate analysis this *bmoR* was also compared to the assembled SAG. When carrying out blastx analysis of the contigs compared to *bmoR* the percent similarity ranged from 33–42%, with a high bit score average of 172. We acknowledge that similarities in this range are low in terms of a reliable annotation as *bmoR*. The discrepancy in the % similarity is likely due to methodological differences (short, unassembled 76 bp reads compared to contigs). Further, the GC content of the contigs upon which *bmoR* was identified was 38%, which is identical to the GC content for the entire SAG assembly (38%). The presence of *bmoR*, similar to *T. butanivorans*, in the *Colwellia* SAG, is not however, diagnostic for presence of the BMO operon. The remaining metabolic pathway used by *T. butanivorans* in butane oxidation was encoded in the SAG (Figure 2) and was highly similar (≥40 bit score) to that of *C. psychrerythraea*. *C. psychrerythraea* does not have a butane monooxygenase thus has a paraphyletic lineage with the *Colwellia* SAG.

The lack of a butane monooxygenase in the SAG may indicate that it, does not, in fact have that metabolic capability. Alternatively, it may be due to insufficient coverage of the genome (30% complete) of the *Colwellia* SAG. To test this hypothesis, we turned to metagenomes from the plume (~0.03 Tb of sequence data) and from sediments (~1 Tb of sequence data), several of which were highly contaminated by the DWH spill (Mason et al., 2012 and 2014, respectively). Although the relative abundance of *Colwellia* in the DWH plume, from which the single cell was retrieved, was low (1–2%) (Mason et al., 2012), the same *bmoR* gene was identified in metagenomes from the plume, as were several additional *T. butanivorans* genes involved in butane oxidation (bit score of ≥40) (Figure 2). However, no butane monooxygenase was recovered in the DWH plume metagenomes. In sediment samples, *Colwellia* had a high relative abundance of 6% in the more contaminated samples (Mason et al., 2014). The higher abundance of *Colwellia* in contaminated sediments (Mason et al., 2014) compared to the deep-sea plume may have resulted in better coverage of *Colwellia*. In the resulting sediment metagenomes, *bmoR, bmoG*, istA, and istB were identified, all of which had a bit score of ≥40, as was the alpha subunit of butane monooxygenase (*bmoX*) (Figure 2). The metagenomic data, particularly in surface sediments, suggested that gaseous hydrocarbon degradation via butane monooxygenase was a plausible pathway for degrading DWH hydrocarbon constituents, however this pathway was not directly linked to *Colwellia*.

Direct evidence for gaseous hydrocarbon degradation by DWH plume *Colwellia*, which were closely related to the *Colwellia* SAG described herein, was provided by Redmond and Valentine (2012) who used SIP to examine the uptake of methane, ethane, and propane. They reported that *Colwellia* enriched from the plume was responsible for the bulk of ^13^C labeled propane and ethane uptake, but also showed, to a far lesser extent uptake of methane by *Colwellia*, consistent with the properties of *T. butanivorans* BMO (Cooley et al., 2009). The putative hydrocarbon degradation pathways discussed above help to resolve several aspects of the microbial response to the oil spill that hitherto were unclear. Specifically, the BMO of *T. butanivorans* is highly efficient at discriminating against methane when grown on ≥C_2_ alkanes (Cooley et al., 2009). At the time *Colwellia* was abundant in mid-June 2010 (Valentine et al., 2010; Redmond and Valentine, 2012), Valentine et al. (2010) reported that, despite the abundance of methane input to the deep-sea during the spill (Joye et al., 2011), propane, ethane (and perhaps butane) fueled microbial respiration. These findings suggested that *Colwellia* was likely involved in degrading gaseous hydrocarbons, but not methane (Valentine et al., 2010), which was substantiated by the aforementioned SIP experiments.

In addition to gaseous hydrocarbon degradation Redmond and Valentine (2012) also reported that *Colwellia* enriched in the DWH plume incorporated benzene in SIP experiments. In the *Colwellia* SAG 4-cresol dehydrogenase (EC 1.17.99.1) and 2-hydroxymuconic semialdehyde dehydrogenase (EC 1.2.1.85) both of which are involved in degradation of several aromatics (e.g., benzene by *Pseudomonas putida* according to Reardon et al., 2000), were identified. These were also identified in the water column and sediment metagenomes. Together, these data support the uptake of aromatics by plume *Colwellia* as reported by Redmond and Valentine (2012).

### Nitrogen

Previously we reported that nitrate was significantly lower in plume samples from which the *Colwellia* SAG was isolated compared to non-plume samples (*p*-value 0.003) (Hazen et al., 2010). One hypothesis is that nitrate was consumed as an electron acceptor during hydrocarbon degradation. As the previously sequenced *C. psychrerythraea* strain was reported to have genes coding for denitrification (Methé et al., 2005), we sought to determine whether the *Cowellia* SAG also possessed the genetic capacity for denitrification. First, a direct comparison was made between *C. psychrerythraea* and unassembled reads from the *Colwellia* SAG that went into the final assembly. This comparison revealed a high sequence similarity (≥40 bit score) of genes involved in denitrification in both the *Colwellia* SAG and *C. psychrerythraea* (Figure 2). For example, *C. psychrerythraea*'s nitrate permease (GI71146856), nitrite reductases (small and large subunits; GI71146751 and GI71143641), nitric-oxide reductase (GI71148260 and GI71144846) and an anaerobic nitric-oxide reductase transcription regulator (GI71282073) were present in the SAG (Figure 2).

Although many genes in the denitrification pathway were common between the *Colwellia* SAG and *C. psychrerythraea*, there were differences. For example, nitrate reduction appears to be encoded by different genes. In the *Colwellia* SAG reduction of nitrate could be carried out via a nitrate/TMAO reductase (COG3005) and a periplasmic nitrate reductase system, NapE component (COG4459). Further an uncharacterized protein involved in response to nitric oxide was present in the SAG (COG3213). The *Colwellia* SAG lacked a nitrous-oxide reductase gene, which may be an artifact of an incomplete genome for the *Colwellia* SAG. It is, however, plausible that the SAG does in fact lack the capability to reduce nitrous oxide. Given the importance of nitrous oxide as a greenhouse gas (Wuebbles, 2009), further elucidating the role of *Colwellia* in denitrifying processes and end-products is necessary, particularly in the context of an unprecedented ecosystem perturbation, such as the DWH oil spill.

### Iron

Baelum et al. (2012) tested the hypothesis put forth by Hazen et al. (2010), that iron could have limited microbial growth, in microcosm experiments with MC252 crude oil. The results suggested that iron limitation resulted in floc formation of which *Colwellia* was the most abundant microorganism. They also reported that in the first 20 days of incubation, lower growth, and hydrocarbon degradation rates were observed, although the total amount of oil degraded after 20 days was the same as in microcosms with sufficient iron. Further, Joung and Shiller (2013) reported that iron concentrations in the plume compared to non-plume samples indicate an enhanced microbial iron demand. This suggests that in the deep-sea plume, at least initially, iron could have affected both the growth and degradation rate of hydrocarbons by *Colwellia*, although is unlikely that iron concentrations were a growth-limiting factor nor a factor in the total amount of hydrocarbons degraded during the spill (Joung and Shiller, 2013).

To better understand *Colwellia*'s iron acquisition strategy we first compared the *Colwellia* SAG to *C. psychrerythraea*, followed by direct annotation of *Colwellia* SAG contigs using CAMERA. Similar to *C. psychrerythraea* the *Colwellia* SAG lacked genes for siderophore production. Yet, highly similar genes when comparing the two (≥40 bit score) were found for iron transport and uptake (Figure 2). Iron(III) ABC transporter proteins (GIs 71277892 and 71143632) and ferrous iron transport proteins (GIs 71144726 and 71143643) were identified, yet the two genomes had different genes coding for interactions with siderophore complexes (Figure 2). Annotation of the *Colwellia* SAG assembly with CAMERA revealed the genome encoded functions involved in iron acquisition, including an outer membrane receptor for the siderophore-iron complex ferrienterochelin (Lundrigan and Kadner, 1986) (COG4771), and an outer membrane receptor for Fe3+-dicitrate (COG4772) (Figure 2). Other functions involved in iron transport were also annotated (COGs 0370, 1629, and 1918) (Figure 2). Taken together, the data suggested that the *Colwellia* SAG could have been at a physiological disadvantage if it was, in fact, unable to synthesize siderophores. Conceivably, *Colwellia* could have relied on scavenging siderophores produced by other microorganisms in the plume for remediating iron limitation. Although we acknowledge that missing genes for siderophore production could be due to the incompleteness of the *Colwellia* SAG, *C. psychrerythraea* also lacks the ability to produce siderophores.

### Floc formation

The *C. psychrerythraea* genome encodes a capsular polysaccharide biosynthesis protein (GI71145258), as does the *Colwellia* SAG (Figure 2). Polysaccharide capsules are found on the cell surface of many bacteria and are involved in, among other processes, adherence of one bacterium to another (Roberts, 1996). Further, *C. psychrerythraea*'s putative polysaccharide biosynthesis glycosyltransferase, which likely functions in extracellular polysaccharide biosynthesis (Methé et al., 2005), was identified in the *Colwellia* SAG. As discussed above, Baelum et al. (2012) observed flocs largely comprised of *Colwellia* that formed under iron limiting conditions in microcosms incubated with MC252 oil. Our findings provide preliminary evidence for a plausible mechanism by which cells could have aggregated together in the face of nutrient, and particularly, iron limitation in the deep-sea plume.

### Chemotaxis and motility

A full suite of genes involved in signal transduction, chemotaxis, and motility were present in the *Colwellia* SAG (Figure 2), many of which were highly similar to *C. psychrerythraea*. For example *C. psychrerythraea*'s chemotaxis proteins CheA, CheY, and CheZ, numerous methyl-accepting chemotaxis proteins (MCP) and the full suite of genes coding for flagellum synthesis and operation were observed in the *Colwellia* SAG. Parales et al. (2000) reported that several strains of bacteria exhibited a chemotactic response to benzene and toluene and suggested MCPs were involved. More direct evidence was provided regarding the role of MCPs in the ability of *Pseudomonas putida* to detect naphthalene (Grimm and Harwood, 1999). Given the suite of genes, including MCP, encoded in the *Colwellia* SAG, it is plausible that the *Colwellia* SAG was able to sense and move toward a chemo-attractant, such as degradable hydrocarbons that accumulated in the deep-sea plume during the DWH spill.

### Adaptation to cold temperatures

Direct comparison of the SAG to the *C. psychrerythraea* genome revealed shared genomic features (≥40 bit score similarity) thought to be involved in adaptation to life at cold temperatures; for example a *C. psychrerythraea* fatty acid cis/trans isomerase, which would allow the cell to alter the ratio of cis- to trans-esterified fatty acids in phospholipids (Methé et al., 2005). The ability to increase cis-isomerization, could enhance membrane fluidity at low temperatures (Methé et al., 2005), which would be an important adaption to life at low temperatures. Further, *C. psychrerythraea*'s putative 3-oxoacyl-(acyl-carrier-protein) reductase and putative 3-oxoacyl-(acyl-carrier-protein) synthase III, which serve key roles in fatty acid and phospholipid biosynthesis (Methé et al., 2005), were identified in the SAG, and could also play a role regulating membrane fluidity at cold temperatures (Russell, 1997).

### Biosynthesis of compatible solutes

The *Colwellia* SAG had genes coding for choline dehydrogenase and betaine aldehyde dehydrogenase, which were highly similar (≥40 bit score) to those of *C. psychrerythraea*. In the genome sequence of the psychrophilic hydrocarbon degrader, *Oleispira antarctica* these were the two mechanisms for biosynthesis of compatible solutes (Kube et al., 2013). Choline dehydrogenase and betaine aldehyde dehydrogenase are involved in catalyzing the reaction that leads to synthesis of the osmolyte betaine (Kube et al., 2013). Genes coding for choline dehydrogenase, and others, were suggested to be involved in synthesis of betaine, which was cited as an osmoprotectant, and also a cryoprotectant in *C. psychrerythraea* (Methé et al., 2005). The dual functionality of an osmoprotectant and cryoprotectant in *Colwellia* would be an important adaptation for the *Colwellia* SAG to life in the low temperature, deep-sea marine environment.

### Comparison of DWH deep-sea plume *Colwellia* SAG to *Oceanospirillales* SAG

Mason et al. (2012) presented an annotated *Oceanospirillales* SAG that was isolated from the proximal plume sample (OV01102/03 in Hazen et al., 2010) from which the *Colwellia* SAG presented herein was also isolated. *Oceanospirillales* had a higher relative abundance in the proximal plume sample compared to *Colwellia* (16S rRNA gene sequence relative abundance was 81% vs. 1% percent, respectively) (Mason et al., 2012). Thus most of the genes and transcripts from the proximal plume sample were thought to derive from *Oceanospirillales* and the role that *Colwellia* played in oil disposition has been largely unexplored. Comparison of the SAGs revealed several differences; namely the dominant hydrocarbon pathways, iron acquisition strategies, and respiration capabilities. First, the *Oceanospirillales* genome encoded cyclohexane degradation by an alkane monooxygenase (Mason et al., 2012), also recently identified in the genome sequence of the closely related *Oleispira antarctica* (Kube et al., 2013). As discussed above, the *Colwellia* SAG encoded *bmoR*, 4-cresol dehydrogenase and 2-hydroxymuconic semialdehyde dehydrogenase, the latter of which are involved in degradation of several aromatics. Thus, the hydrocarbon constituents potentially degraded by these two gammaproteobacteria, that increased in abundance in the plume, are disparate. Second, the ability to synthesize siderophores to acquire iron, which may have affected hydrocarbon respiration rates early on (but was not likely limiting) by the microbial community in the deep-sea plume during the oil spill, would have given the dominant *Oceanospirillales* a competitive advantage over *Colwellia*. As discussed above, the *Colwellia* described here may have been able to scavenge siderophores produced by other microorganisms, perhaps those of *Oceanospirillales*, to acquire iron. Finally, the genome of *Oceanospirillales* did not encode a pathway for denitrification, in contrast to the *Colwellia* presented here. Therefore, when faced with reduced oxygen concentrations *Colwellia* could have continued to carry out respiration, while *Oceanospirillales* may not have been able to do so.

### Comparison of DWH deep-sea plume metagenomes and metatranscriptomes and sediment metagenomes to the *Colwellia* SAG

Mason et al. (2012) reported relative abundances of 1–2% of *Colwellia* in metagenomes from the proximal plume closest to the DWH wellhead (1.8 km away) and at a more distant location, referred to as the distal plume (10.8 km away), collected May 26-June 2, 2010. Of these metagenome sequence reads 0.30% mapped to the *Colwellia* SAG. To determine if the *Colwellia* SAG was active in the deep-sea plume, 5S, 16S, and 23S rRNA sequences were subtracted from the plume metatranscriptomes. These reads were then mapped against the assembled *Colwellia* SAG. Comparison of the *Colwellia* SAG to proximal plume station metatranscriptome sequences revealed that 0.20% of the metatranscriptome reads mapped to the single-cell assembly. Fewer metatranscriptome reads from the distal station mapped to the *Colwellia* SAG (0.01%). Thus a representative of plume *Colwellia* may have been actively degrading gaseous and simple aromatic hydrocarbons, albeit at low levels, in late May 2010, when these hydrocarbons were less abundant. In contrast Rivers et al. (2013) reported that 16% of their metatranscriptome reads from two DWH plume stations (6–8 km from the wellhead, collected May 26-June 3, 2010; at the same time we sampled) mapped to *C. psychrerythraea*, although these authors used blastx while we used Bowtie2. Further, the abundance of *Colwellia* was significantly higher in Rivers et al. (2013; see Figure 1B for abundance) 16S rRNA gene survey as compared to the samples presented in Mason et al. (2012).

Previously, we characterized the microbial community in 64 surface sediment samples, which had varying degrees of hydrocarbon contamination from the DWH spill (Mason et al., 2014). As discussed above, *Colwellia* was up to 6% (relative abundance) of the microbial community in these sediments, particularly those with the highest hydrocarbon concentrations (Mason et al., 2014). From these samples 14 were selected for metagenomic sequencing. Of these 14 samples, 7 were determined *a priori* to exceed the Environmental Protection Agency (EPA)'s PAH benchmarks for aquatic life. The *Colwellia* SAG assembly was mapped to the sediment metagenomes with 0.25–0.04% of reads mapping. The most SAG reads mapped to 6 of the 7 samples that exceeded the EPA's PAH benchmarks. These results are similar to the plume metagenome and indicate that the *Colwellia* SAG sequenced was represented in both the water column and sediments that were contaminated during the DWH spill.

## Conclusion

Analyses of the microbial community response to the oil spill revealed a clear story of microbial succession, however, few detailed descriptions of the cellular physiology of the indigenous microbes that responded to the hydrocarbon inputs have been presented. Herein we describe several aspects of a *Colwellia* single-cell genome that furthers our understanding of the potential role that *Colwellia* played during microbial succession in the deep-sea hydrocarbon plume that formed during the DWH spill. At the time that *Colwellia* was most abundant in the plume, methane was highly abundant but largely not degraded in favor of other gaseous hydrocarbons. This could have been due to the genetic capacity of the *Colwellia* to preferentially degrade gaseous hydrocarbons at the expense of methane, if it does, in fact, have a butane monooxygenase. Additionally, genes involved in BTEX degradation in the *Colwellia* SAG were identified. These preliminary findings may help to resolve the simultaneous increase in *Colwellia* abundance when these hydrocarbon compounds accumulated in the plume. The *Colwellia* SAG was captured in metagenomes from the water column and surface sediments that were contaminated by the DWH spill. In addition, both *Oceanospirillales* and *Colwellia* SAGs possessed genes for chemotaxis and motility, suggesting that this is an advantage for rapid response to hydrocarbon inputs in the deep-sea. We also provide preliminary evidence that the *Colwellia* species described herein may have been able to produce polysaccharides for floc production that corresponds to the high abundance of flocs in microcosms under iron limited conditions as reported by Baelum et al. (2012). This could represent a novel property enabling *Colwellia* to cope with conditions of low iron concentrations, but further experiments are necessary to confirm this hypothesis.

## Author contributions

Olivia U. Mason participated in the research cruise during which the deep-sea plume sample for single-cell isolation and sequencing was obtained. Olivia U. Mason carried out bioinformatics analyses and wrote the manuscript. James Han carried out bioinformatics analyses and helped write the manuscript. Tanja Woyke carried out single-cell genome isolation and sequencing and helped write the manuscript. Janet K. Jansson conceived the experimental design and helped write the manuscript.

### Conflict of interest statement

The authors declare that the research was conducted in the absence of any commercial or financial relationships that could be construed as a potential conflict of interest.
