# Supplementary material for: Single-cell genomics reveals features of a Colwellia species that was dominant during the Deepwater Horizon oil spill
Source: Front Microbiol. 2014 Jul 8;5:332. doi: 10.3389/fmicb.2014.00332 (PMC4085564; doi:10.3389/fmicb.2014.00332)
Supplement: Supplementary file 1 [file DataSheet1.PDF]

Supplementary Materials

Supplementary Results

Quality control
The *Colwellia* SAG assembly GC content was unimodal with an average of 38 +/- 3%, consistent with the GC content of *Colwellia psychrerythraea* 34H (Genbank ID CP000083). Using all single cell contigs, we ran blastn (blast+ version 2.2.28, parameters: -task megablast -perc_identity 90 -evalue 1e-30 -dust yes -num_threads 1 -outfmt '6 qseqid sseqid salltitles bitscore evalue length pident qstart qend qlen qframe sstart send slen sframe') analysis against all microbial genomes in the NCBI refseq database (database downloaded 2013/05/30). All contigs >100bp alignment length blast hits were to *Colwellia* with the exception of a single contig, which matched a possible laboratory contaminant (*Ralstonia*). This suggested that the assembly likely contained sequence data from a single genome and was free of major contamination.
